# Vector-Borne Pathogens in Guard Dogs in Ibadan, Nigeria

**DOI:** 10.3390/pathogens12030406

**Published:** 2023-03-02

**Authors:** Isabella Gruenberger, Amelie-Victoria Liebich, Temitayo Olabisi Ajibade, Oluwasola Olaiya Obebe, Nkiruka Fortunate Ogbonna, Licha N. Wortha, Maria S. Unterköfler, Hans-Peter Fuehrer, Adekunle Bamidele Ayinmode

**Affiliations:** 1 Department of Pathobiology, Institute of Parasitology, University of Veterinary Medicine, 1210 Vienna, Austria; 2 Department of Veterinary Parasitology and Entomology, Faculty of Veterinary Medicine, University of Ibadan, Ibadan 200132, Nigeria

**Keywords:** vector-borne diseases, dogs, *Hepatozoon*, *Babesia*, *Trypanasoma*, *Anaplasma*, tick-borne diseases, blood parasites

## Abstract

Canine vector-borne diseases are of great relevance not only regarding animal welfare but also in relation to the One Health concept. Knowledge concerning the most relevant vector-borne pathogens in dogs is scarce and limited to stray dogs in most western African regions, and there is virtually no information about the situation in kept dogs presenting (regularly) to vets. Therefore, the blood samples of 150 owned guard dogs in the Ibadan area—in the southwest of Nigeria—were collected and analyzed for the DNA of Piroplasmida (*Babesia*, *Hepatozoon*, *Theileria*), Filarioidea (e.g., *Dirofilaria immitis*, *Dirofilaria repens*), Anaplasmataceae (e.g., *Anaplasma*, *Ehrlichia*), Trypanosomatidae (e.g., *Leishmania*, *Trypanosoma*), *Rickettsia*, *Bartonella*, *Borrelia* and hemotropic *Mycoplasma* using molecular methods. Overall, samples from 18 dogs (12%) tested positive for at least one pathogen. *Hepatozoon canis* (6%) was the most prevalent blood parasite, followed by *Babesia rossi* (4%). There was a single positive sample each for *Babesia vogeli* (0.6%) and *Anaplasma platys* (0.6%). Moreover, one mixed infection with *Trypanosoma brucei/evansi* and *Trypanosoma congolense* kilifi was confirmed (0.67%). Generally, the prevalence of vector-borne pathogens in this sample group of owned dogs in southwest Nigeria was lower than in prior studies from the country and in other parts of Africa in total. This leads to the assumption that, firstly, the exact geographical location has a major influence on the incidence of vector-borne diseases, and, secondly, it seems to make a difference if the dogs are owned and, therefore, regularly checked at a veterinary clinic. This study should raise awareness of the importance of routine health check-ups, tick and mosquito prophylaxis, and a well-managed infectious disease control program to prevent vector-borne diseases in canines.

## 1. Introduction

Canine vector-borne diseases (VBDs) caused by various helminths, protozoa, or bacteria play a major role in the wellbeing of dogs worldwide. Furthermore, various canine VBDs are of relevance for human health because of the zoonotic potential of some pathogens [1]. The importance of gaining knowledge of the agents and the diseases they are causing has an ever-increasing significance relating to globalization, increased human and animal traffic, and climate change [2]. Vector-borne pathogens (VBPs) can be transmitted by diverse blood-feeding arthropods, including ticks, mosquitoes, phlebotomine sandflies, fleas, and lice [3]. Among all of these, ticks have the highest relevance—for instance, the incidence of tick infestation in owned dogs was reported to be 71.2% in Nigeria, with *Rhipicephalus sanguineus* having the highest prevalence, followed by a large margin by *Haemaphysalis leachi* [3,4,5,6]. The most important VBPs occurring in Nigeria are *Hepatozoon canis*, *Babesia rossi,* and *Ehrlichia canis*, with *H. canis* being the most prevalent pathogen. Additionally, *Babesia vogeli*, *Anaplasma platys*, *Mycoplasma haemocanis*, *Candidatus* Mycoplasma haematoparvum, *Theileria* sp., and *Trypanosoma* sp. have been detected using molecular diagnostic tools, with a lower prevalence [7,8,9].

However, in Nigeria, studies based on highly sensitive PCR techniques are limited [3,10]. Furthermore, most of the existing studies have focused solely on the existence of piroplasmids or tick-borne diseases, and extensive data on the prevalence of mosquito-borne pathogens is rather limited.

This study was conducted to provide a comprehensive overview of the prevalence of a wide range of VBPs in owned dogs in Nigeria. Therefore, dogs presented at the Veterinary Teaching hospital of Ibadan in southwest Nigeria were screened for the presence of the DNA of Piroplasmida (e.g., *Babesia, Hepatozoon, Theileria*), Filarioidea (e.g., *Dirofilaria immitis, Dirofilaria repens*), Anaplasmataceae (e.g., *Anaplasma*, *Ehrlichia*), *Trypanasomatidae* (e.g., *Leishmania*, *Trypanosoma*), *Rickettsia*, *Bartonella*, *Borrelia,* and hemotropic *Mycoplasma* using molecular diagnostic tools.

## 2. Results

Of the 150 dogs sampled, 57 were female, 65 were male, and 28 were of unknown sex; 65 were older than one year, and 57 were estimated to be younger than one year. Overall, 18 (12%) dogs tested positive for at least one vector-borne pathogen (Table 1).

*Hepatozoon canis* was the most predominant parasite, with 9 (6%) of the animals testing positive in PCR assays. Sequence analyses showed two different genotypes with no common occurrence of both (seven Type 1 and two Type 2).

PCR assay targeting the 18S rRNA gene of *Babesia* spp. tested positive in seven samples, which was confirmed as *Babesia rossi* in six samples (prevalence of 4%) and one as *Babesia vogeli* (prevalence of 0.6%) at sequence analysis.

DNA of *Anaplasma platys* was determined in one sample (0.67%).

After initial PCR for the presence of the DNA of *Trypanosoma* spp., one sample tested positive. An additional PCR technique was performed, amplifying within the ribosomal RNA ITS-1 region [11]. With this technique, a mixed infection (0.67%) with *Trypanosoma brucei/evansi* (430 bp) and *Trypanosoma congolense* kilifi (560 bp) was detected.

Within this study, the DNA of *Leishmania* spp., *Theileria* spp., *Ehrlichia* spp., *Rickettsia* spp., *Bartonella* spp., *Borrelia* spp., and haemotropic *Mycoplasma* was not detected.

All dogs positive for *Babesia* spp. presented with pyrexia (inner body temperature of >39 °C), with two dogs even showing a highly increased temperature (>41 °C).

Generally, there was no significant correlation between pyrexia and testing positive for one of the VBPs (Table 2).

Other documented clinical symptoms of VBP-positive dogs were very diverse, showing a poor body condition score (2/18), anorexia (8/18), and fever (10/18) as the most common symptoms. There was no documentation available for 4 of the 18 infected dogs.

Regarding the age of the positive animals, more than half (7/12) were puppies or subadults (<1 year). Statistics showed that neither age (*p*-value = 0.4073), poor body condition (*p*-value = 1), nor sex (*p*-value = 0.5851) correlated with an infection.

## 3. Discussion

The purpose of this study was to investigate the distribution of canine VBPs in owned dogs in southwest Nigeria using molecular methods. A regular survey of the current situation regarding the prevalence of parasites in domestic animals is of great importance to ensure the right diagnosis, treatment, and overall good public health in relation to zoonosis. Compared to already existing data from Nigeria and other African countries, the results showed a lower prevalence concerning all pathogens tested for: 12% in total. It should be noted that the study presented here from Ibadan, Nigeria, did not involve street dogs but dogs kept as pets that received regular veterinary check-ups. Therefore, the results of this study can only be interpreted for this sub-population of dogs.

Street dogs, in contrast, usually have poor nutritional status and lack basic health care, such as deworming, vaccinations, or treatment with antiparasitics. They often spend the night outdoors and are at great risk of being infested by vectors [12,13,14].

Previous studies in Nigeria have shown wide variability in the prevalence of VBPs ranging between 4.4% and 59.1%. The high heterogeneity regarding the prevalence can be explained mainly due to pooled sample groups and different diagnostic methods. Most of them are based on microscopic detection, which is less sensitive and more subjective than molecular methods, and where various infections are simply not found or misdiagnosed more easily [3,10]. This could be the reason for the completely different results in studies that only used microscopy [15,16]. However, the main obstacle to using molecular approaches for epidemiological studies in Nigeria is the sparse availability of the necessary facilities due to their high cost, which makes them mostly out of reach for most epidemiologists [17].

Nevertheless, PCR also has its limitations—e.g., if the pathogen burden in the blood remains below the diagnostic level [10]. Furthermore, sample size and the studied group, and season vary in different studies.

A related study conducted seven years earlier, in 2013, at Ibadan Veterinary Teaching Hospital, showed 76.7% positive samples, which is significantly higher than in the present study [10]. The reason for this big gap was that the researchers pooled all samples for suspected cases of hemoparasitism (e.g., high temperature, anemia, hemoglobinuria), in contrast to this field study where every dog that presented at the clinic within the time frame was included—regardless of the symptoms shown—in the study. Under these circumstances, the study was able to achieve realistic prevalence numbers under field study conditions. The comparison of these two similar studies with completely different results shows the huge difference the sample group makes and that you cannot compare prevalence if the study was not carried out under the same circumstances.

The main reason for the lower percentages of pathogens found in dogs in this study could be due to the fact that all dogs examined were owned dogs, which are more likely to regularly receive medical treatment at a veterinary clinic, and they had an overall good body condition, with only 16 dogs presenting in poor condition. Many studies that included stray dogs or semi-owned dogs in their sample group demonstrated a much higher prevalence of pathogens in total, as well significantly higher numbers in free-roaming dogs than in owned dogs [18,19].

The total prevalence of VBPs appeared to be higher in the northern and central regions of Nigeria than in the south and southwest [3]. Higher numbers in the north may be due to the following reasons: The first is the climate, which shows warmer temperatures all year round and a more humid climate than in other provinces [20,21]. It has been proven that warmer temperatures correlate positively with the circulation and spread of vectors [22]; The second explanation can be found in the socio-political circumstances caused by the Islamist terrorist group Boko Haram, which dominates the north and does not allow an effective vector and pathogen control program [23].

The lower total prevalence found in this study concurs with the results of prior studies from the southwestern part of Nigeria [3,24,25]. It can be concluded that there seems to be a strong connection between the geographical location of sampling in Nigeria and the number of positive cases. However, the present study is the first study of owned dogs in Nigeria, and direct comparison to studies from the northern and central regions is therefore limited.

The results suggest a low prevalence of VBDs in the southwest of Nigeria, and their occurrence in the dry season can even be estimated to be lower, as samples were collected during the rainy season, which in this part of the country, lasts from May to October, with a slight peak from June to August [26]. The seasons with higher rainfall and humidity are more favorable for ticks and their development [27].

The predominant pathogen in Nigeria seems to be *H. canis*, with a total retrospective prevalence found to be 20.3% [25], 41.4% [28], 16.9% [3], and 17.3% [15]. Not only in this western African country but in other sub-Saharan countries, *H. canis* appears to be the most important pathogen overall, reaching a prevalence of 58.6% in the latest survey that collected data from six different countries all over the continent [29]. The presence of a pathogen is directly related to the presence of a competent vector. The tick with the highest prevalence in Nigeria is *R. sanguineus*, a tick species that is found globally from tropical and subtropical to Mediterranean areas. With a percentage of over 90% in tick samples collected from dogs in Nigeria, it is not surprising that *H. canis* is the most frequently found pathogen, with *R*. *sanguines* being the primary vector; this may also explain the occurrence of *Babesia* species [3,4,30,31]. In southern Africa, *Haemaphysalis leachi* is known as a vector for *B. rossi*; however, due to the absence of a high tick infestation with *H. leachi* in Nigerian dogs, *R. sanguineus* plays a potential role as a vector of *B. rossi* in this African country. After all, the local vector for *B. rossi* is still being discussed [4,31].

*Babesia rossi* was detected less often in this study, with an overall prevalence of 4.0%, compared with previous studies from Obeta et al. [30], Amuta et al. [18], Okubanjo et al. [15], Opara et al. [16], and Jegede et al. [32], reporting 10.8%, 10.2%, 17.3%, 57.1%, and 8.9%, respectively.

Only 1 out of 150 dogs tested positive for the DNA of *Babesia vogeli*, correlating with the similarly low numbers seen in past studies [5,24,28]. The lower rate of infections with *Babesia vogeli* compared to *Babesia rossi* can be explained by its geographical distribution. Whereas *B. vogeli* is more often found in tropical, subtropical, and Mediterranean climate zones, *B. rossi* is assumed to be limited to sub-Saharan Africa, of which Nigeria is a part [31,33].

There was one dog positive for the genetic material of *Anaplasma platys*. The number of studies examining the prevalence of this pathogen in western Africa is limited; however, two previous studies conducted in Nigeria reported similarly low numbers, at 6.6% [28] and 5% [5]. In comparison to the available data from other African countries (South Africa, Egypt), there seems to be no substantial difference in the distribution of the microorganism, with the prevalence ranging between 4.4% and 13.3% [34]. *Anaplasma platys* was frequently identified in tropical regions, which suggests that its distribution is dependent on a humid tropical climate.

Interestingly, we could not detect the DNA of *Ehrlichia canis* in any of the sampled dogs. Our results were contrary to a previous study conducted in southwest Nigeria, where *E. canis* DNA was documented in 22.9% of the tested pet animals without any exclusion criteria [35]. A prevalence of 12.7% of *E. canis* was documented in the northern parts of Nigeria, with the significant difference being that this study only included patients with a tick infestation or showing symptoms of tick-borne diseases [28].

The use of peripheral blood for the diagnosis of *Leishmania* spp. might be a reason for the absence of the *Leishmania* DNA. The most accurate and precise way to diagnose *Leishmania* is PCR from the bone marrow, lymph nodes, spleen, or skin [36,37,38]. Whole blood, buffy coat, and urine have a much lower PCR sensitivity than the previously stated tissues [36,39]. The two biggest risk factors for dogs getting leishmaniasis are lifestyle and exposure to sandflies [40].

Only a small number of studies using PCR have reported *Trypanosoma* spp. occurrence in dogs in Nigeria [3,10,41,42]. This constituted a positive case in this study, which was confirmed as a mixed infection with *T. brucei/evansi* and *T. congolense* kilifi, which is especially concerning. Therefore, the monitoring of the current infestation with the *Trypanosoma* subspecies of domestic animals—and especially of pet dogs living close to humans—should not be disregarded, considering the high risk of zoonosis of some subspecies of *Trypanosoma*.

The absence of microfilaremic dogs with *Dirofilaria immitis* and *D. repens* was not in line with the findings of Ombugadu et al. [43], Kamani and González-Miguel [44] and Ogbaje and Abel-Danjuma [45], which showed a relatively high incidence. The reason for this could be related to the usage of deworming drugs, such as ivermectin, praziquantel, and levamisole, in 58 cases of the tested individuals. The active ingredients mentioned are also effective for *Dirofilaria* spp. and its larvae [46]. Ivermectin exhibits very good action against microfilariae and larval tissues during the first six weeks of *D. immitis* infection, despite being ineffective against the adult stages of *D. immitis* [47,48,49].

The low number of VBPs and negative results for *Dirofilaria*, *Leishmania*, *Bartonella,* and *Mycoplasma* in our study were due to the high level of treatment of the domestic and working dogs included in this study. Many of the animals were pre-treated with various deworming agents, and the majority of dogs showed no disease symptoms and were generally in good health.

The clinical presentation of the dogs with babesiosis in this study seemed to be more moderate, showing high fever more often than in other documented cases in Nigeria, where the clinical presentation has been described as mild or even sub-clinical [5]. Regarding the presenting symptoms, it must be considered that the symptoms of hemoparasitism are often nonspecific and depend on the immune status and age of the animal. Therefore, they can vary substantially [50]. This could also be supported by the statistical tests that have been carried out, which showed no relationship between various symptoms and carrying a VBP. The low correlation between the clinical presentation and a positive VBD sample can be explained further by the fact that many other (often chronic) infectious diseases (for instance, parvovirosis, leptospirosis, etc.), which were not considered in this study, can play a significant role regarding the health status of dogs in developing countries. They can also result in nonspecific symptoms such as pyrexia and anorexia [51,52].

To be able to draw a reliable conclusion regarding the clinical disease, it is necessary to expand the study and focus on the clinical symptoms in a more comprehensive setting, including, for example, laboratory examinations noting tick infestation and using blood serology, in addition to PCR testing to diagnose the disease.

## 4. Materials and Methods

### 4.1. Collection and Specification

Between June and August 2020, blood samples were collected from 150 dogs at the University of Ibadan’s Veterinary Teaching Hospital and a private veterinary clinic in Ibadan (7°23′ N, 3°53′ O), Nigeria. Within this study, domestically owned dogs who were regularly presented at the veterinary clinic (not street dogs) were included. These were dogs of both sexes, ranging in age from five weeks to eighteen years, mainly serving as pets or guard dogs. Sex, age, weight, symptoms, inner body temperature, body condition, deworming history, and the latest medical treatment were documented whenever possible. Blood was collected from the cephalic vein, and 50 µL was transferred to filter paper (VWR International GmBH, Vienna, Austria), dried by air, and labeled. The samples were collected and transferred to the Institute of Parasitology at the University of Veterinary Medicine in Vienna, where they were kept dry in pressure bags until further processing.

### 4.2. DNA Extraction

DNA was extracted from 4 × 4 mm cutouts of blood-soaked filter paper impregnated with PBS, using InstaGene™ Matrix (Bio-Rad, Vienna, Austria), as reported previously [53].

### 4.3. Pathogen Detection

Conventional PCRs were mainly used in this study [54,55,56,57,58,59,60]. Piroplasmida and Trypanasomatida were additionally screened for using nested PCR, and Filarioidea was evaluated using touchdown PCR. The additional nested PCR protocol (ITS) for Trypanasomatida was necessary because, after the first screening within the 18S rRNA region, the segment could only be evaluated as positive, but the sequence did not allow species determination. The additional PCR was carried out with new primers (TRYP3/TRYP4 -> TRYP1/TRYP2; [11]) for the final detection of various *Trypanosoma* (sub)species/variants (e.g., *T. congolense* savannah, *T. congolense* forest, *T. congolense* kilifi, *T. brucei/evansi*, *T. vivax*). PCR was carried out with a volume of 25 μL using 5X Green Reaction Buffer and GoTaq^®^ G2 Polymerase (5 U/µL; Promega, Walldorf, Germany) and was run in the “Eppendorf Mastercycler^®^ nexus” and “Eppendorf Mastercycler^®^ ep gradient S”. PCR products were analyzed by electrophoresis on 1.8% agarose gels stained with Midori-Green Advance^®^ (Biozym, Hessisch Oldendorf, Germany). Afterward, the positive samples were sequenced by LGC Genomics GmbH (Berlin, Germany), and sequences were compared for similarity using the basic local alignment search tool (BLAST) in GenBank^®^ (http://www.ncbi.nlm.nih.gov/BLAST). Obtained sequences were uploaded to GenBank^®^ (accession numbers: OP837316-OP837332, OP837527). 

### 4.4. Statistics

For statistical analysis, prop.test was used for the comparison of equal or given proportions, working with R version 4.2.0 (R Foundation for Statistical Computing, Vienna, Austria).

## 5. Conclusions

This study proves the presence of various VBPs in owned guard dogs in Nigeria. Nevertheless, the relatively low prevalence of VBDs in this study group of owned dogs in contrast to street dogs showed that regular vet appointments appear to have a positive effect on the distribution of infectious diseases and, moreover, their possible transmission to humans. This highlights the significant importance of routine health check-ups, vector prophylaxis, and a well-managed infectious disease control program. For further studies, it would be of great interest to look at a comparison group of street dogs exclusively to investigate whether they are exposed to more potential pathogens and to extend the study to include comprehensive clinical examinations.

## Figures and Tables

**Table 1:** Vector-borne pathogens in owned dogs in Ibadan (no detailed data are available for four of the positive dogs. Among those, three tested positive for *Hepatozoon canis* (Type 1), and one tested positive for *Babesia canis rossi*).

Pathogen	Sex	Age	Breed	Body Condition	Temperature (in °C)	Symptoms
*Babesia rossi*	M	3 months	German Shepard	Poor	41.1	Anorexia, apathy, emesis
*Babesia rossi*	F	4 years	Alsatian	Good	41.1	Anorexia, diarrhea
*Babesia rossi*	M	4 months	Boerboel	Good	39.7	Anorexia, apathy, anemia
*Babesia rossi*	M	3 years	Mixed	Good	39.8	Anorexia, ectoparasites
*Babesia rossi*	F	4 years	German Shepherd	Good	39.3	Splenomegaly
*Babesia vogeli*	M	4 years	Boerboel	Good	39.7	None
*Hepatozoon canis* (Type1)	F	3 months	Caucasian	Good	38.8	Swollen jaw
*Hepatozoon canis* (Type1)	F	5 months	Caucasian	Good	40.0	Anorexia, swollen jaw
*Hepatozoon canis* (Type2)	M	5 months	Alsatian	Good	39.8	None
*Hepatozoon canis* (Type2)	F	9 months	Alsatian	Good	39.2	Anorexia
*Hepatozoon canis* (Type1)	M	4 months	Alsatian	Good	39.8	Alopecia
*Hepatozoon canis* (Type2)	F	1.5 years	Rottweiler	Good	38.5	Mange
* Trypanosoma brucei * and *Trypanosoma congolense* kilifi	F	5 months	Boerboel	Good	38.5	None
*Anaplasma platys*	M	6 months	Alsatian	Poor	41.2	Anorexia

**Table 2:** Results of correlation testing between detection of vector-borne pathogens and fever, body condition, age, and sex. Note that *p*-values are >0.05, and results were therefore not considered statistically significant.

	*p*-Value	Confidence Interval	Odds Ratio
Fever	0.2845	−0.0932412 to 0.4888456	2.403672
Body condition	1	−0.2210936 to 0.2096370	0.9916495
Age	0.4073	−0.1599416 to 0.4907108	0.5197553
Sex	0.5851	−0.4337743 to 0.1983246	0.6289681

## Data Availability

Not applicable.
